# The effect of preoperative suggestions on perioperative dreams and dream recalls after administration of different general anesthetic combinations: a randomized trial in maxillofacial surgery

**DOI:** 10.1186/1471-2253-15-11

**Published:** 2015-01-28

**Authors:** Judit Gyulaházi, Katalin Varga, Endre Iglói, Pál Redl, János Kormos, Béla Fülesdi

**Affiliations:** Department of Anesthesiology and Intensive Care, Medical and Health Science Centre, University of Debrecen, Debrecen, Hungary; Department of Affective Psychology, Institute of Psychology, Eötvös Loránd University, Budapest, Hungary; Department of Applied Mathematics and Probability Theory, Faculty of Informatics, University of Debrecen, Debrecen, Hungary; Department of Oral and Maxillofacial Surgery of the University of Debrecen, Debrecen, Hungary; Department of Economic Analysis and Business Informatics, Faculty of Economics and Business Administration, University of Debrecen, Debrecen, Hungary

## Abstract

**Background:**

Images evoked immediately before the induction of anesthesia with the help of suggestions may influence dreaming during anesthesia.The aim of the study was to assess the incidence of evoked dreams and dream recalls by employing suggestions before induction of anesthesia while administering different general anesthetic combinations.

**Methods:**

This is a single center, prospective randomized including 270 adult patients scheduled for maxillofacial surgical interventions. Patients were assigned to control, suggestion and dreamfilm groups according to the psychological method used. According to the anesthetic protocol there were also three subgroups: etomidate & sevoflurane, propofol & sevoflurane, propofol & propofol groups. Primary outcome measure was the incidence of postoperative dreams in the non-intervention group and in the three groups receiving different psychological interventions. Secondary endpoint was to test the effect of perioperative suggestions and dreamfilm-formation training on the occurrance of dreams and recallable dreams in different general anesthesiological techniques.

**Results:**

Dream incidence rates measured in the control group did not differ significantly (etomidate & sevoflurane: 40%, propofol & sevoflurane: 26%, propofol & propofol: 39%). A significant increase could be observed in the incidence rate of dreams between the control and suggestion groups in the propofol & sevoflurane (26%-52%) group (p = 0.023). There was a significant difference in the incidence of dreams between the control and dreamfilm subgroup in the propofol & sevoflurane (26% vs. 57%), and in the propofol & propofol group (39% vs.70%) (p = 0.010, and p = 0.009, respectively). Similar to this, there was a significant difference in dream incidence between the dreamfilm and the suggestion subgroups (44% vs. 70%) in the propofol & propofol group (p = 0.019). Propofol as an induction agent contributed most to dream formation and recalls (χ2-test p value: 0.005). The content of images and dreams evoked using suggestions showed great agreement using all three anesthetic protocols.

**Conclusion:**

The psychological method influenced dreaming during anesthesia. The increase of the incidence rate of dreams was dependent on the anesthetic agent used, especially the induction agent.

The study was registered in ClinicalTrials.gov. Identifier: NCT01839201.

**Electronic supplementary material:**

The online version of this article (doi:10.1186/1471-2253-15-11) contains supplementary material, which is available to authorized users.

## Background

Among others, the most important components of general anesthesia is providing a sufficient level of hypnosis during the procedure, as well as reducing anxiety in the perioperative period. Perioperatively used hypnosis and suggestions may be employed, in addition to local, or general anesthesia as complementer techniques for anxiolysis, sedation, relaxation, pain alleviation, and amnesia [1–6]. Previous reports showed that suggestions administered in the preoprative period may shorten hospital lenght of stay, may result in decreased pain intensity and reduced opioid requirements in the postoperative setting [2, 7–9].

In recent decades it has beeen proven that despite the use of depth of anesthesia monitors the occurrance of perioperative dreams cannot be avoided [10–13]. Unpleasant perioperative dreams or dream recalls may lead to decreased patient satisfaction related to the surgical/anesthesiological event and thus should be reduced.

It seems that imagination guided by suggestions before induction of anesthesia may modify dream recalls after recovery. The main goal of suggestive techniques in the perioperative phase is to turn the content of dreams toward a favourable direction that is considered a pleasant event by the patient. So far little attention has been paid to the administration of perioperative psychological methods that may meet these requirements.

Along these lines, in the present study we intended to assess whether dream recalls can be infuenced by two different psychological methods administered in the preoperative setting.

We intended to answer the following study questions:

1. What is the incidence of spontaneous dreams and recallable dreams while using different general anesthesiological methods?
2. What is the effect of perioperative suggestions and dreamfilm-formation training on the occurrance of dreams and recallable dreams in different general anesthesiological techniques?
3. What is the influence of induction and maintenance agents on the psychological methods?
4. Finally we intended to assess whether a relationship exists between the content of the preoperatively administered psycholotherapeutical method and the postoperatively recalled dreams.

## Methods

The investigations were carried out between 2009 and 2012 by the anaesthesia team of the Department of Anesthesiology and Intensive Care at the Oral and Maxillofacial Surgery ward of the Faculty of Dentistry, University of Debrecen, in a prospective, randomized fashion.

Ethics: Ethical approval for this study (Ethical Committee N° DEOEC RKEB/IKEB 2830–2008) was provided by the Ethical Committee University of Debrecen, Hungary (Chairperson József Szentmiklósi MD, Nagyerdei krt. 98. Debrecen. Phone: +3652411600).

Adult patients undergoing elective maxillofacial surgery were included, with whom verbal communication was possible. After an informed consent, written agreement was obtained from all patients. Exclusion criteria were: mental retardation, tracheotomy, and inability to communicate (See CONSORT checklist in Additional file 1).

### Grouping of the patients

Patients were randomly allocated into three groups according to the following aspects:

-  *In the control group* spontaneous dreams of patients were assessed under anesthesia without suggestions.
-  *In the suggestion group* patients received suggestions evoking their images exclusively in the operating theatre at the time of induction. For this, patients were instructed to find out and fix a favourite place "where they want to travel" during anesthesia.
-  *In the "dreamfilm group"* the patients worked out a dreamfilm-plan using the favourite place technique one day prior to surgery. At induction, the series of images prepared by suggestions was evoked.

In all three of the previously listed groups 3 further subgroups were formed based on the anesthesiological technique used:

-  *Subgroup 1:* anaesthetic induction with etomidate (0,15-0,3 mg/kg), maintenance with sevoflurane (1 MAC, low-flow tchnique),
-  *Subgroup 2:* anesthetic induction with propofol (1,5-2,5 mg/kg), maintenance with sevoflurane (1 MAC, low-flow tchnique),
-  *Subgroup 3 (TIVA group):* anesthetic induction with propofol (1,5-2,5 mg/kg), maintenance with propofol (8–10 mg/kg/hour).

**Figure 1:**
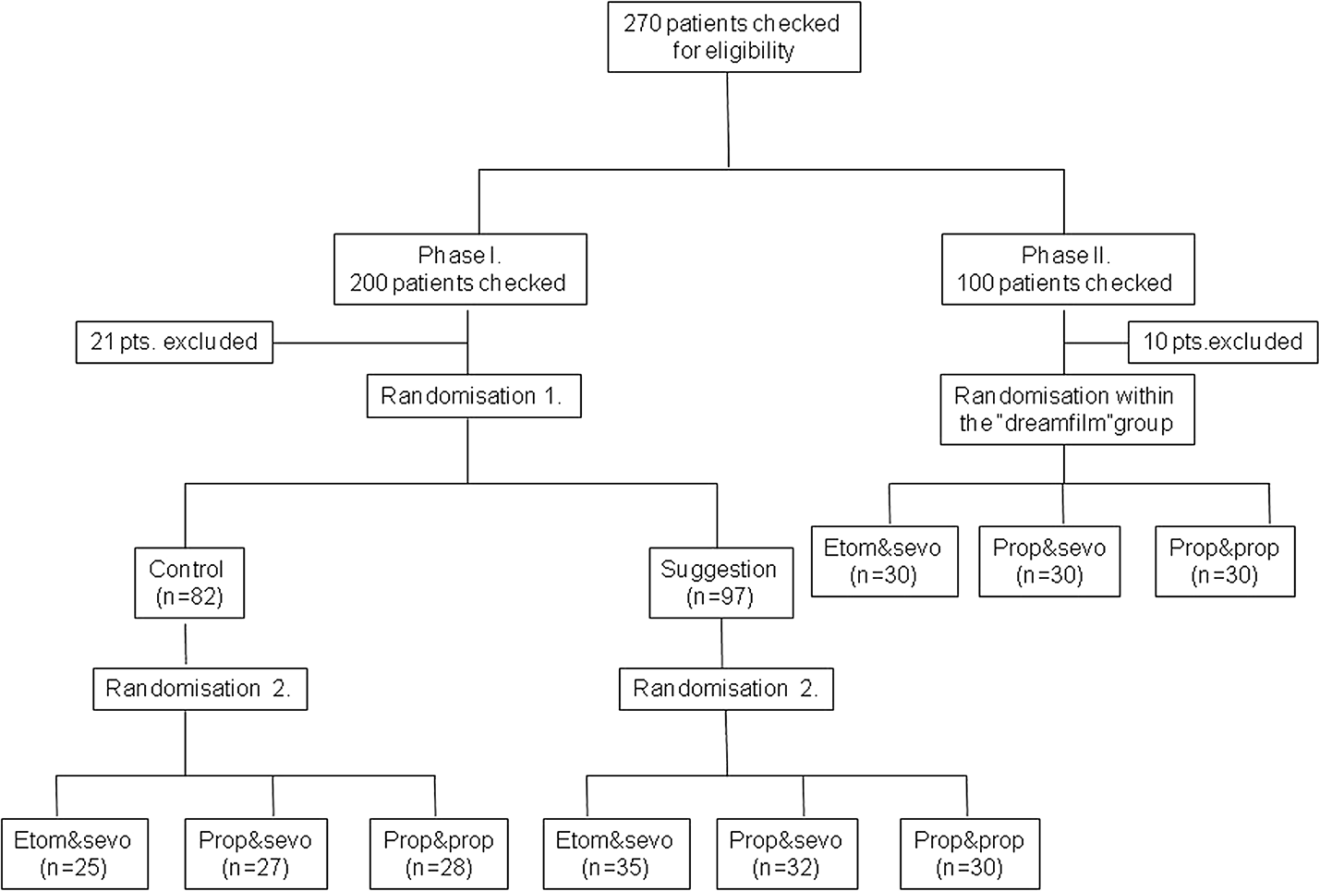
**Inclusion of patients and randomisation procedure.**

Because of methodological reasons, the investigations were perfomed in two phases. As we intended to exclude the possibility that patients of dreamfilm groups might communicate during the preoperative day and therewith might influence the results of the psychological method, in the first phase only control, (no suggestions were administered) and suggestion group patients (suggestions administered in the OR) were included. Randomisation in this first phase meant main grouping and selecting the anesthesiological subgroup. In this phase of the study 60 patients were allocated per every anesthetic method. Envelope randomisation was carried out in the operating theatre, immediately before induction, the patients were allocated to the suggestion and the control groups, respectively (a total of 180 patients). Investigations in phase two were separated from phase one in time. The people taking part in the study were not selected, all patients presenting at the department in the given time period were included in the dreamfilm group provided that they met the selection criteria and who did not refuse participation. Again, envelope randomisation occurred in the operating theatre to chose the general anesthetic technique. Using three anaesthetic protocols, this amounted to 3×30 subjects. Arrangement of the experiments is summarized in Figure 1.

### Psychological methods used

The psychological methods used for inducing hypnosis were modifications of those used and described earlier by Faymonville et al. in detail [14].

*The "favourite place" technique"* describes guided imagination of life events with the help of positive suggestions immediately before induction of anesthesia. In the operating theatre the patient was informed about what was going to happen, what sensation the induction agent would cause and was also told that the waking stimulus would be their name. We asked the patient not to pay attention to noises, only to what the anesthetist said. The suggestion technique itself starts with a relaxation exercise, using suggestions promoting calm, deep breathing and muscle relaxation. The patient is not simply asked to remember an event, the aim is to produce a feeling that they are "virtually" in their favourite place. Meanwhile the patient is involved in the imagination process in a dialogue form.

*"Dreamfilm method"*: Patients were met one day prior to surgery and were asked to imagine a film that they would like to "watch" during the anesthesia. Thus, in this case a "favourite place" is produced by the patients, featuring in the prepared dreamfilm. This film is prepared one day prior to surgical anesthesia. Anesthesiologists evoke the previously prepared dreamfilms with suggestions administered at the time of anesthetic induction.

The main difference between the "favourite place" and the "dreamfilm" group was that in the latter group patients were working on elaborating the dreamfilm one day before surgery. In both groups, the favourite place and the dreamfilm that was produced by the patient were recorded prior to anesthesia by the physician for the sake of further analysis, i.e. patients were asked to recall them verbally. All suggestions and anesthesias were performed by the same person (JGY), who is a certified and experienced anesthetist and psychotherapeutist.

*Postanaesthetic management in the OR:* After the patients were awakened, they were called by their names, and were informed where they were and that the operation had been finished. Thereafter they received amnesia-lifting suggestions, they were asked, before recovery of full consciousness, to retain their dreams and recall them so that later in the ward they could report them to the independent assistants. At this phase, all events related to the recovery period were recorded, including the patient’s first reactions during the early recovery phase.

### Gathering data

The patients were interviewed about their dreams and the postoperative questionnaires were filled by the department’s assistants, 10 and 60 minutes after recovery, respectively. They were pretrained, independent (blind) staff personnel who were not aware of the grouping status of the patients. The postoperative questionnaire contained parameters of the patients’ general condition: blood pressure, pulse, complications, and communication. A pivotal part of this questionnaire were questions about the dream report in the postoperative setting. One section of the questionnaire concerned the assessment of the relationship between the anesthetist and the patient (rapport) as well as of the team’s work and the patient’s anxiety level related to the procedure.

### Anesthetic and monitoring techniques

General anesthesia as well as the suggestion techniques for patients in groups 2 and 3 were applied by a single physician (JGy). Midazolam (7,5- 15 mg) and atropin (0,5-1 mg) were administered per os one hour before anesthetic induction as premedication in all patients. Induction and maintenance of anesthesia was performed depending on the grouping status of the patients, as decribed above. In all three anesthetic protocols, pain relief was achived with fentanyl (0,02-0,05 mg/kg boluses), muscle relaxation with atracurium (0,5 mg/kg bolus, 0, 15mg/kg rep.), or with mivacurium (0,2 mg/kg), depending on the length of surgery. Intratracheal intubation was performed in all cases, followed by a pressure controlled ventilation tecnhnique, using oxygen-air mixture, with Dräger Primus anaesthetic device. Monitoring was secured using an Infinity Kappa XLT monitor: as part of standard monitoring, non-invasive blood pressure, pulse oxymetry, capnograpy, ECG, and relaxometry were performed. Anesthesia was managed to ensure that hypnotic depth measured by BIS monitoring was between 40 and 60 throughout the entire time elapsed between intubation and wound closure. Monitoring started at the time point before induction of anesthesia and ended after total recovery of the patient, awake state of consciousness and return of adequate communication were reached. *Postoperative analgesia:* Tramadol (4×1 mg/kg) and metamizole (4x0,5-1 g) were used to reduce postoperative pain as was necessary for proper pain relief. Analgesia and anxiolysis measurements: The efficiency of analgesia was graded every hour by the patients based on the rating scale used in the Hungarian school assessments (5 being the best grade = no pain, 4 = mild pain, 3 = moderate pain, 2 = strong pain, and 1 = worst, intolerable pain).

### Statistical methods

The statistical analysis was performed by SPSS 11.5. We used the following procedures and tests:

-  χ^2^-test for independence of two variables, provided by the SPSS Crosstabs procedure.
-  *T*-test for independent samples
-  One-sample binomial test.

### Dependent variables examined

Patient report 10 and 60 minutes respectively after recovery about the appearance of a dream (yes/no).

## Results

The most important confounding factors and anamnestic data are summarized in Table 1. There was a marked female dominance (female: male ratio = 169:101). The majority of the patients were between 19 and 75 years of age. The occurrence of spontaneous dreams in the sample was almost 3/week on average, among them almost half were repeated and generally recalled. When assessing previous history of anesthesia, dreaming occurred in less than 10% of the patients and 2/3 of these dreams were recallable. General anesthesia lasted for 85.5 ± 56.4 minutes (means ± SD) and the bispectral index was 41.37 (range 0–59), indicating proper level of hypnosis.

**Table 1:** **Confounding factors and preoperative anamnestic data**

Sample size		270
Sex	Female	169 (62.6%)
	Male	101 (37.4%)
Age distribution	11-18yr	20 (7.4%)
	19-30yr	91 (33.7%)
	31-50yr	78 (28.9%)
	51-75yr	79 (29.3%)
	75 < yr	2 (0.7%)
Frequency of dreaming per week at home	Mean (±SD)	2.78 (±2.17)
Repeated dreams	Yes	122 (45.2%)
	None	148 (54.8%)
Recalled home dreams	Generally recalled	135 (50%)
	Sometimes recalled	89 (33%)
	Non-recalled	41 (15.2%)
	No dreams at all	5 (1.9%)
Present indication of surgery	Accident	78 (28.9%)
	Cancer	70 (25.9%)
	Inflammatory	14 (5.2%)
	Reconstructive	35 (13%)
	Other	73 (27%)
Level of preoperative anxiety	1 (weak)	10 (3.7%)
	2	21 (7.8%)
	3	96 (35.6%)
	4	74 (27.4%)
	5 (strong)	69 (25.6%)
History of general anesthesia	Yes	158 (58.5%)
	No	112 (41.5%)
Experience by former anesthesia	Neutral	73 (46.2%)
	Positive	59 (37.3%)
	Negative	26 (16.5%)
Dream during former anesthesia	Yes	12 (7.6%)
	No	146 (92.4%)
Recalled dream during former anesthesia	Yes	8 (66.7%)
	No	4 (33.3%)

## The incidence of spontaneous dreams during general anesthesia in the control group

In general, spontaneous dreams during general anaesthesia were reported in 35% of our cases (n = 28 out of 80 patients). In a second step, we analysed the number of reported perioperative dreams according to the general anaesthetic technique. It has been found that dreams were reported in 40% (n = 10) in the etomidate & sevoflurane group, 26% (n = 7) in the propofol & sevoflurane group and 39% (n = 11) in the propofol & propofol group. Pearson chi-squared test indicated no significant difference in the reported dreams among the three general anaesthesia technique subgroups (p = 0.478) indicating that spontaneous dreams have similar incidence independent of the anaesthetic technique. When assessing whether the patients are able to recall the content of their dreams in the postoperative setting, it is worth mentioning that although Pearson chi square test indicated no significant difference between incidence of dream recalls in the control group (p = 0.27), recallable dream/all dream ratio was gradually higher in the propofol & propofol group (74%), than in the etomidate & sevoflurane and propofol & sevoflurane groups (50% and 42% respectively).

### Assessment of the impact of different perioperative psychotherapeutical interventions (preoperative suggestions and "dremafilm" method) on the incidence of dreams and dream recalls

When we compared the incidence of dreams in the control, suggestion and "dreamfilm" groups according to the anaesthetic technique the following results were found:

*Etomidate & sevoflurane group:* The incidence of reported dreams is similar irrespective of the fact whether no psychotherapeutic intervention was administered, suggestions or "dreamfilm" method was applied (Pearson chi-square p = 0.883).

*Propofol & sevoflurane group and propofol & propofol groups:* In contrast to this, as indicated by the statistical analysis the incidence of reported dreams depended on the perioperative psychological intervention (Pearson chi-square p = 0.046 for propofol & sevoflurane and p = 0.038 for propofol & propofol groups, respictively), suggesting that these anesthesia techniques may precipitate dream formation in combination with psychotherpeutic interventions Table 2.

**Table 2:** **Testing the homogeneity of distributions of dreaming using Pearson chi-square test results grouped according to anesthetic protocols**

		Not dreaming	Dreaming	p-value
		% within row		
Etomidate/sevoflurane	Control	60.0%	40.0%	.883
	Suggestion	55.9%	44.1%	
	Dreamfilm	53.3%	46.7%	
	Total	56.2%	43.8%	
Propofol/propofol	Control	60.7%	39.3%	.038
	Suggestion	56.3%	43.8%	
	Dreamfilm	30.0%	70.0%	
	Total	48.9%	51.1%	
Propofol/sevoflurane	Control	74.1%	25.9%	.046
	Suggestion	48.5%	51.5%	
	Dreamfilm	43.3%	56.7%	
	Total	54.4%	45.6%	

*Subgroups analysis whithin the same anaesthetic groups:* Based on the results of the previous statistical results we performed a secondary subgroup analysis within the different general anaesthesia groups. The results are summarized in Table 3. This subgroup analysis proved again that administration of propofol both in combination with sevoflurane or as a part of total intravenous anaesthesia results in a significantly higher incidence of dream reports if a perioperative psychotherapeutic intervention is applied. The most powerful effect was observed when a combination of propofol was used with the "dreamfilm" method. Administration of propofol both as an induction agent and also used for maintenance led to an increased ability of the patients to recall their dreams in the majority of the subgroups (Table 3).

**Table 3:** **Pairwise comparison of dreams and recallable dreams in the different anesthesia technique groups**

Etomidate & sevoflurane group				
	**All dreams**		**Recallable dreams**	
**Control – suggestion**	40% vs. 44%	p = 0.37	20% vs. 32%	p = 0.15
**Control – dreamfilm**	40% vs. 47%	p = 0.31	20% vs. 30%	p = 0.20
**Suggestion – dreamfilm**	44% vs. 47%	p = 0.42	32% vs. 30%	p = 0.42
**Propofol & propofol group**				
	**All dreams**		**Recallable dreams**	
**Control – suggestion**	39% vs. 44%	p = 0.36	29% vs. 44%	p = 0.116
**Control – dreamfilm**	39% vs. 70%	p = 0.009	29% vs. 63%	p = 0.004
**Suggestion – dreamfilm**	44% vs. 70%	p = 0.019	44% vs. 63%	p = 0.063
**Propofol & sevoflurane group**				
	**All dreams**		**Recallable dreams**	
**Control – suggestion**	26% vs. 52%	p = 0.02	11% vs. 39%	p = 0.007
**Control – dreamfilm**	26% vs.57%	p = 0.009	11% vs. 53%	p < 0.001
**Suggestion – dreamfilm**	52% vs. 57%	p = 0.34	39% vs. 53%	p = 0.137

### Testing the effect of the induction and maintenance agents on dreaming probabilities among the three subgroups

We also intended to clarify whether drugs used for induction or rather those used for maintenance influence the effectivity of our psychological methods. For this purpose we merged propofol & propofol and propofol & sevoflurane into one group and reran the χ^2^-test for homogeneity of the dreaming distributions among the three psychological method groups. The p-value of Pearson chi-square tests was p = 0.005. If we make a comparison with the etomidate/sevoflurane group, where the Pearson chi-square tests p-value was p = 0.883, one may conclude that propofol as an induction drug significantly advances the effect of our psychological methods compared to etomidate. For the sake of consistency we repeated the previous test to investigate the role of the maintenance drug. We merged the first and third anesthetic protocol groups (etomidate & sevoflurane and propofol & sevoflurane) into one group, and repeated the χ^2^-test. The p-value of the Pearson chi-square test for etomidate & sevoflurane was p = 0.107, while it was 0,038 in propofol & propofol group. Hence, propofol, also as a maintenance drug, significantly advances the effect of our psychological methods, while sevoflurane does not.

In all fairness, comparison of the p-values p = 0.005 and p = 0.038 also shows that the change is more significant in the case propofol is taken into account as an induction agent. We may also conclude that for the effectivity of our psychological methods the induction drug is a more important factor than the maintenance drug.

### Relationship between the content of preoperative imaginations and perioperative dreams

In the final analysis the independent observer made a comparison of the preoperative imaginations and the postoperatively reported content of the dreams. The connection of the content of the suggested image and that of the dream were 94.7% in the suggestion group and 83% in the dreamfilm group across the three anesthetic protocols. When we estimated the probability of connection separately for each narcotic protocol, but merging the suggestion and the dreamfilm groups into one group, we found that in the etomidate & sevoflurane group 86%, in the propofol & sevoflurane group 90%, in the propofol & propofol group 88% of preoperative imaginations and postoperatively reported dreams corresponded to each other.

## Discussion

In the present study we found that sponaneous dreams may be observed in approximately one third of patients undergoing general anesthesia, independent of the anesthetic method. The second finding of our observations is that the incidence of dreams and dream recalls are more frequent in those patients in whom preoperative suggestions are applied before and during induction. Furthermore, formation of dreams and dream recalls are dependent on the anesthetic technique. Finally, we observed that the content of dreams recalls can be guided by psychological methods with a probability of at least 90% and they increase recallable dream ratio independently from anesthetic method used.

The mode of action of the psychological methods demonstrated in our study corresponds to the "Tetris phenomenon" described previously by Stickgold et al. While studying the effect of practising the Tetris game on NREM dreams, Stickgold et al. found that the Tetris game appeared in about 60% of the subjects’ dreams during the next two nights [15]. The anesthetic state may have similarities compared to NREM sleep and the neural correlates of the two states show great similarities [16–20]. Thus, it can be assumed that, via a similar mechanism, consolidation of episodic memory and dream formation may occur during anesthesia, too.

Perioperative dreams and dream recalls are regular and unavoidable events of general anesthesia. According to the literature, the incidence of perioperative dreams varies between 1% to 57% [13, 20, 21]. There are data to support that the occurrance of dreams depends on the anesthetic drug used [13, 20], and there are some studies which do not [12]. In the present study we could not prove any difference between the rates of spontaneous dreams while using different general anesthetic combinations (etomidate/sevoflurane; propofol/sevoflurane; propofol/propofol). It has to be mentioned that this was also true for dream recalls in the anesthetic subgroups without psychological intervention. In contrast to this, the use of different psychological methods contributed to an increase of dreaming incidence that was dependent on the anesthetic protocol employed, predominantly if propofol was used as an induction agent.

It has been suggested that during NREM sleep memory consolidation takes place simultaneously with the appearance of fast sleep spindles [22, 23]. It is highly remarkable that during the induction of propofol anesthesia several authors have demonstrated sleep spindle activity [24–26]. Murphy et al. found that, in propofol induction, gamma- power almost doubled at loss of consciousness, indicating lively cognitive activity [18]. Moreover, Breshears detected the presence of coupled theta-gamma oscillations during propofol induction and recovery alike [26]. All this may explain why propofol induction allows good memory consolidation from before induction. During anesthesia if hypnotic depth decreases temporarily, the events potentially perceived by the patients may be incorporated into their dream in a new context [12]. However, if intraoperative hypnosis is managed properly, perioperative dreams may be the result of episodic memory consolidation of events immediately preceding anesthesia.

Based on our results two sets of clinically important considerations can be drawn. First: environmental stimuli in the operating theatre may be incorporated into perioperative dreams during general anesthesia. One has to remember that due to their altered state of consciousness, patients are capable of producing very vivid imagery while suggestibility increases [5]. Therefore, care should be taken especially during the induction phase to reduce annoying and unpleasant acoustic or visual stimuli. It is the whole OR team’s responsibility to provide a quiet environment around the patient in order to avoid unpleasant dream formations. The second important consideration is rather methodological. For a long time, perioperative dreams were considered as events that are indicators of inappropriate depth of anesthesia that should be reduced to a minimum during daily clinical practice [27]. The present study has allowed us to form a significantly different point of view. As we have proved, similarly to others, perioperative dreams cannot be avoided during clinical practice, they can even be increased by administering preoperative suggestions. We are of the opinion that dreams occurring during anesthesia should, in fact, be turned in a favourable direction by choosing proper induction agents and through the administration of pleasant suggestions during induction of anesthesia. Our voice, our gestures and behaviour are all suggestions that unwittingly evoke images, emotions, which may influence the patient’s dreams during anesthesia and their experiences at recovery. In a recent systematic review Wobst stated that even patients wo do not reach the stage of hypnotic trance may benefit from hypnotic suggestions [1]. Thus, anesthetists should in some way act as a psychotherapists during the induction phase of general anesthesia.

## Conclusions

We strongly believe that, besides providing a calm environment in the OR, all anaesthetists should work out a method that provides pleasant suggestions to the patients because favourable perioperative dreams may contribute to patient satisfaction related to the anesthetric event and therefore efforts should be made to do it. In a previous meta-analysis it has been suggested that hypnosis may function via changes in patients’expectancies for outcomes and adjunctive hypnosis is beneficial in 89% of surgical patients [28].

## Electronic supplementary material

Additional file 1: CONSORT 2010 checklist of information to include when reporting a randomised trial*. (DOCX 36 KB)
